# Reward-based modulation of task-switching performance: a diffusion model analysis

**DOI:** 10.3758/s13414-023-02711-7

**Published:** 2023-06-01

**Authors:** Timo Weber, Kerstin Fröber, Stefanie Schuch

**Affiliations:** 1 Institute of Psychology, RWTH Aachen University, Aachen, Germany; 2 Department of Psychology, University of Regensburg, Regensburg, Germany

**Keywords:** Motivation, Reward, Cognitive control, Task inhibition, N-2 repetition costs

## Abstract

Investigating the interface between motivation and cognitive control, we conducted two task switching experiments (*N* = 96 each) with reward manipulation where participants switched between three different tasks. We measured N-2 task repetition costs, which denote the performance decrement in N-2 task repetition sequences (ABA) relative to N-2 task switch sequences (CBA), and which are presumed to be a marker of inhibitory control in task switching. Participants in the reward group received performance-contingent reward in the second phase of each experiment, and in the second experiment they were additionally penalized for errors. Reward thresholds were determined individually based on participants’ performance during the first phase of each experiment. Participants in the control group did not receive any reward. The reward manipulation led to faster performance in the reward group relative to the control group. Diffusion modeling revealed that the reward manipulation induced an increase in drift rate parameter, consistent with dopamine-based enhancement of attentional focus under reward. Contrary to our expectations, no robust evidence for a reward-based modulation of N-2 repetition costs was found across the two experiments. N-2 task repetition costs were small in both experiments, and possibly, a larger amount of inhibitory control is needed in order to obtain empirical evidence for a reward-related modulation thereof. However, additional analyses suggested that reward may not interact with inhibitory control on the task level at all.

## Introduction

Motivation and cognitive control have been suggested to be closely linked (for reviews, see Botvinick & Braver, 2015; Braver, 2015; Chiew, 2021; Chiew & Braver, 2011; Dreisbach & Fischer, 2012; Kouneiher et al., 2009; Parro et al., 2018; van Steenbergen et al., 2019; Yee & Braver, 2018). For instance, Botvinick and Braver (2015) defined cognitive control as “the set of superordinate cognitive functions that encode and maintain a representation of the current task (…)” (p. 84), and motivation as “the orienting and invigorating impact, on both behavior and cognition, of prospective reward” (p. 84). They suggested that cognitive control can be conceptualized as reward-based decision-making. In line with this notion, many studies have manipulated motivational state by introducing performance-contingent reward in the experimental paradigm.

A considerable body of evidence suggests that performance-contingent reward leads to enhanced recruitment of cognitive control: Interference effects in conflict tasks become smaller when a reward manipulation is introduced, suggesting that the influence from the irrelevant stimulus feature is attenuated (e.g., Padmala & Pessoa, 2011); performance in stop-signal tasks is improved upon reward (e.g., Boehler et al., 2014; Leotti & Wager, 2010; Padmala & Pessoa, 2010), and proactive control is increased by reward prospect in the AX continuous performance task (Chiew & Braver, 2013, 2014; Fröber & Dreisbach, 2014, 2016a; Hefer & Dreisbach, 2016, 2017, 2020; Locke & Braver, 2008) and the dual-task paradigm (Fischer et al., 2018). Furthermore, task-switching performance becomes better under reward (e.g., Aarts et al., 2010; Hippmann et al., 2019; Kleinsorge & Rinkenauer, 2012; Nieuwenhuis & Monsell, 2002).

For instance, in a recent task-switching study with TMS stimulation, Hippmann et al. (2019) had participants switch between two number categorization tasks (parity task and magnitude task) and manipulated reward expectancy on a trial-by-trial basis: A reward cue was presented before each stimulus, indicating the amount of money that could be gained in the following trial (“1 cent” or “7 cents”). They observed the standard finding of slower reaction times (RTs) in task switch trials than task repetition trials, and these task switch costs were smaller in trials where high reward was expected (about 15 ms) as compared to trials where low reward was expected (about 30 ms). That is, switching between tasks became easier (i.e., was associated with lower costs) when reward expectancy was high, and this effect was independent from stimulation site. In error rates, a similar data pattern of reduced switch costs under high reward expectancy was observed, but only with stimulation over the left inferior frontal junction (IFJ), not with stimulation at a control site, suggesting that the left IFJ plays a causal role in the reward-based modulation of task switch costs.

Several (voluntary) task switching studies furthermore showed that performance-contingent reward affects task-switching performance differently depending on the immediate reward history (Fröber et al., 2018, 2019, 2020; Fröber & Dreisbach, 2016a, 2021; Shen & Chun, 2011). The prospect of a high reward generally enhanced performance in terms of decreased RT compared to low reward prospect, but the same high reward prospect either reduced or increased switch costs and increased or reduced voluntary switch rates depending on the reward sequence: Task switches benefitted more from increasing reward expectation (smaller switch costs and increased voluntary switch rates when reward increased from low to high in the current trial), whereas task repetitions benefitted more from remaining high reward prospect (higher switch costs and reduced voluntary switch rates when reward remained high).

Not only reward sequence, but also task sequence seems to be a modulating factor on the impact of reward on task-switching performance. In another recent task-switching study that measured behavioral performance and EEG, Zhang et al. (2016) let participants switch between three different tasks (two number categorization tasks: parity task, magnitude task, and one very simple task that required pressing both possible response keys simultaneously) and measured N-2 repetition costs, which occur when comparing performance in the last trial of a N-2 task repetition sequence (e.g., ABA) and the last trial of a N-2 task switch sequence (e.g., CBA, where A, B, and C denote different tasks). N-2 repetition costs are usually thought to reflect persisting inhibition of a previously abandoned task that needs to be overcome when switching back to this task (see Koch et al., 2010, for review). Other than Hippmann et al. (2019), Zhang et al. (2016) manipulated reward in a between-subjects design. Participants in the control group never received any reward, while participants in the reward group could receive a reward on every trial, depending on their performance (they received 2 cents for each correct response). Zhang et al. (2016) observed smaller N-2 repetition costs in the reward group (13 ms, 4.4% more errors) than in the control group (70 ms, 4.3% more errors), suggesting that reward modulates inhibitory control in task switching. This behavioral effect was accompanied by a reward-related modulation of N-2 repetition costs in the N2 ERP component, and source localization revealed that the N2 effect originated from the anterior cingulate cortex (ACC), a brain region that has been associated with another component of cognitive control, namely conflict monitoring processes (Botvinick et al., 2004). Moreover, Zhang et al. (2016) observed faster but more error-prone overall performance in the reward group than control group, suggesting that the reward manipulation might have induced a shift towards a less cautious response strategy.

Reward-based modulations of N-2 repetition costs have also been investigated in a study by Jiang and Xu (2014). They let participants switch between three different tasks (letter, digit, symbol classification) and manipulated reward in a within-subjects design: Participants started with a baseline phase without reward, and then proceeded to a reward phase. During the reward phase, the possibility of receiving performance-contingent reward varied on a trial-by-trial basis (as in Hippmann et al., 2019), with a reward cue appearing prior to each stimulus and indicating whether or not a reward could be gained in the upcoming trial (incentive vs. no-incentive cues). In Jiang and Xu’s (2014) data, N-2 repetition costs did not differ between baseline and reward phase, and neither between incentive and no-incentive trials within the reward phase. Because Jiang and Xu (2014) had expected to find reward-based modulation of N-2 repetition costs, they performed follow-up analyses looking at local aftereffects of reward on a trial-by-trial basis. They observed that N-2 repetition costs differed as a function of the incentive cue presented in the N-1 trial, with larger N-2 repetition costs when an incentive cue had been presented in the N-1 trial than when a no-incentive cue had been presented. Based on these results, they suggested that inhibition of the just-performed task in trial N-2 is increased when a reward can be gained in trial N-1, and the aftereffect of this increased inhibition is then measured in trial N. In other words, they observed a reward-based modulation of N-2 repetition costs, with larger N-2 repetition costs when reward expectancy in the N-1 trial was present than when it was not.^1^

Hence, the evidence for reward-based modulation of N-2 repetition costs is mixed: While Zhang et al. (2016) reported smaller N-2 repetition costs when reward was possible (in the reward group) than when it was not (control group), Jiang and Xu (2014) did not observe any difference in N-2 repetition costs between reward phase and control (baseline) phase. Instead, when zooming into trial-by-trial effects during the reward phase, they observed an increase in N-2 repetition costs when reward had been possible in the N-1 trial than when it had not.

### The present study

In the present behavioral study, we aimed to further investigate reward-based modulations of N-2 repetition costs. In Experiment 1, we used two different task switching paradigms that had produced reliable N-2 repetition costs in previous studies. One paradigm consisted of three number categorization tasks (parity task, magnitude task, interval task), where participants had to classify digits as odd or even, smaller or larger than five, or positioned in the inner or outer areas of a mental number line. The other paradigm consisted of three face categorization tasks (age task, gender task, eye color task), where participants had to categorize faces as young or old, female or male, or with bright or dark eye color. In Experiment 2, we used a paradigm where participants had to categorize pictures of household items as belonging to kitchen or garage, standing upright or upside down, or being smaller or larger than a shoebox. In all paradigms, we used the same two response keys (left and right) for responding. The response sets for the different tasks were overlapping (i.e., the same two response keys served for responding to the three different tasks), which is considered to produce maximal interference between the different tasks and evoke reliable N-2 repetition costs (see Koch et al., 2010, for review).

In both experiments, we manipulated reward between-subjects (control group vs. reward group), as well as within-subjects: Participants in the reward group started with a baseline phase without reward, and then proceeded to a reward phase; participants in the control group did not receive any reward in either phase. In the reward phase, every trial could potentially be rewarded, depending on the participant’s performance. Individual RT thresholds were computed based on baseline performance, and participants received reward feedback if they responded correctly and if their RT was within the fastest third of their RT distribution in the baseline phase. In the reward phase of Experiment 2, incorrect trials were penalized (see *Method* for details).

In a first step, we analyzed global effects of the reward manipulation, comparing the baseline and reward phases in the reward and control groups. A difference in N-2 repetition costs between baseline and reward phase in the reward group could be due to reward effects, and/or to practice effects, while such a difference in the control group could only be due to practice effects. Hence, if we observed a larger modulation of N-2 repetition costs from baseline phase to reward phase in the reward group than in the control group, we would interpret this as globally reward-induced modulation of N-2 repetition costs.

In a second step, we analyzed local effects of the reward manipulation in the reward phase of the experiment. Following Jiang and Xu (2014), we analyzed N-2 repetition costs as a function of reward in the N-1 trial. To this end, we separated the data according to response speed in the N-1 trial (fast vs. slow). In the reward group, but not in the control group, the fast N-1 trials had been followed by reward feedback. In slow N-1 trials, no reward feedback had been presented in either group. Hence, if we observed a larger modulation of N-2 repetition costs by N-1 response speed in the reward than control group, we would interpret this effect as locally reward-induced modulation of N-2 repetition costs.

In addition to analysis of mean performance (RTs and error rates), we applied diffusion modeling to the present data. The diffusion model is a relatively simple computational model to account for performance in speeded choice-RT tasks (Ratcliff, 1978; Voss et al., 2013; for reviews, see Ratcliff & McKoon, 2008; Ratcliff et al., 2016). It complements the analysis of mean performance and provides a richer account of the cognitive processes underlying a particular data pattern, by additionally taking the shape of RT distributions into account. In its simplest form, the model assumes that overall RT in a single trial can be split up into a decisional and a non-decisional component. The decisional component reflects the choice (i.e., response selection) process; the non-decisional component encompasses all cognitive processes before and after response selection (e.g., stimulus encoding, motor processes). The choice process can be described by the average rate of evidence accumulation towards one or the other response alternative (drift rate) and the amount of evidence required before a decision is made (boundary separation). The simple diffusion model, thus, provides three parameters: drift rate, boundary separation, and non-decision time.

N-2 repetition costs are mainly reflected in the drift rate parameter, with smaller drift rate in the last trial of an ABA than CBA sequence (Schuch, 2016; Schuch & Grange, 2019; Schuch & Konrad, 2017), in line with the idea that response selection in the last trial of an ABA sequence is more difficult due to persisting inhibition of the N-2 task. We expected to find this effect in the present study as well. Moreover, we expected any reward-based modulations of N-2 repetition costs, inasmuch as they are due to modulations of task inhibition, to be reflected in drift rate as well.

The reward manipulation might also have effects on diffusion model parameters that are independent from N-2 repetition costs. For instance, given that Zhang et al. (2016) observed faster but more error-prone performance in the reward condition than control condition, it is possible that the reward manipulation induced a shift towards a less cautious response strategy, which would be reflected in a lower boundary separation in the reward condition. Moreover, it has been suggested in the literature that reward enhances cognitive control (for review, see Botvinick & Braver, 2015), by improving the signal-to-noise ratio of task-relevant representations, which is mediated on the neurophysiological level by the dopamine system (e.g., Aarts et al., 2010; Durstewitz & Seamans, 2008; Yee & Braver, 2018). Such an effect of reward-based improved signal-to-noise ratio of cognitive representations would be reflected in the diffusion model drift rate, with enhanced drift rate in the reward condition.

To summarize, we expected reward to (a) modulate the effect of N-2 repetition costs in drift rate, and (b) lead to overall smaller boundary separation and/or higher drift rate in the reward condition than in the control condition.

## Experiment 1

### Method

#### Participants

In total, 97 participants were tested. Forty-eight participants performed the digit-categorization tasks and were tested in Aachen; they were students, or friends of students, of RWTH Aachen University. Half of the participants were randomly assigned to the control group (18 female, six male; mean age = 23.3 years, *SD* = 3.6, range 18–35 years), the other half to the reward group (17 female, seven male; mean age = 25.0 years, *SD* = 7.4, range 18–54 years). The other 49 participants performed the face-categorization tasks and were tested in Regensburg; they were students, or friends of students, of Regensburg University. Twenty-four participants were randomly assigned to the control group (20 females, four males, mean age = 20.0 years, *SD* = 3.2, range 19–32); 25 to the reward group (24 females, one male, mean age 22.1 years, *SD* = 6.2, range 17–46). All participants received either partial course credits or money (8€ per full hour) for participation. With a total sample size of 96 participants, a power analysis with MorePower v6.0.4 (Campbell & Thompson, 2012) revealed a power of .68 to detect a medium-sized (${\mathrm n}_p^2$ = .06) three-way interaction of motivation group, phase, and task sequence.

#### Tasks, stimuli, and responses

In the digit-categorization paradigm, the digits 1–9 (except 5) served as stimuli, and were presented in white on black background, centrally on the screen. Participants switched between three possible tasks: categorizing the digit as smaller or larger than five (size task), odd or even (parity task), or situated in the inner (digits 3, 4, 6, 7) or outer (digits 1, 2, 8, 9) area of a mental number line ranging from 1 to 9 (interval task).

In the face-categorization paradigm, eight different pictures of faces were used as stimuli (354 ×472 pixels, presented centrally on the screen). They were taken from the face database described in Schuch et al. (2012). There was one facial picture for each of the eight possible combinations of young/old, female/male, and bright/dark eye color. Participants switched between the three possible tasks of categorizing the face as young or old (age task), female or male (gender task), or having bright (i.e., blue-green) or dark (brown) eyes (eye color task).

In both paradigms, the task for each upcoming trial was indicated by a colored frame presented centrally on the screen, which was then followed by the stimulus (digit or facial picture, respectively) presented inside the frame. In the digit categorization paradigm, a red frame indicated the size task, blue the parity task, and yellow the interval task. In the face categorization paradigm, a red frame indicated the age task, blue the gender task, and yellow the eye color task.

A left and right response key served for responding in both paradigms (the keys Y and M on a QWERTZ keyboard). Participants were instructed to use their left and right index fingers for responding. The eight possible response mappings were fully counterbalanced across participants in each sample. A reminder summarizing the individual response mappings for the three tasks was placed below the screen and remained visible throughout the experiment.

The trial procedure was identical for both paradigms. Every trial started with the presentation of a task cue (frame) for 500 ms, which was followed by the presentation of a stimulus (digit or facial picture, depending on paradigm). Cue and stimulus stayed on the screen until one of the response keys was pressed. Upon key press, a performance-dependent feedback message was presented for 1,000 ms (see below). Then, the screen remained empty for 500 ms (for 1,000 ms after error) before the next trial started.

#### Reward manipulation

In the control group, participants performed the task switching experiment (four blocks of 120 trials each) without any reward manipulation. In the reward group, participants performed the baseline phase (first half: two blocks of 120 trials each) without any reward manipulation; in the reward phase (second half), they could achieve points for fast and correct performance, and every trial in the reward phase could potentially be rewarded. The criterion for a “fast response” was determined based on individual performance in the baseline phase (block 2 only); thresholds were computed separately for each participant, task, and task sequence. A point was achieved when RT was within the fastest third of the RT distribution for that participant, task, and task sequence.

Participants in the reward group received the following instruction (in German) after the baseline phase: “For the remainder of the experiment, you can achieve points. All participants will participate in a competition. The person with the highest number of points will be awarded an Amazon gift card worth 15€. The person who comes second will receive a gift card worth 10€, the person who comes third a gift card worth 5€.” The first, second, and third prizes were determined separately for the Aachen and Regensburg samples; the participants with the highest, second-highest, and third-highest number of points at each site received their prizes after data collection was completed.

All participants received performance-dependent feedback after each trial during the task switching experiment. In the control group, participants received the message “Correct!” after correct responses, and “Error” after incorrect responses (all messages were presented in German). This was also the case for participants in the reward group during the baseline phase. In the reward phase, the reward-group participants received one of the following feedback messages: “Correct! +1 Point” for correct and fast responses; “Too slow! No point” for correct but slow responses, or “Error! No point” for incorrect responses.

#### Procedure

Participants were tested in individual sessions that lasted for about 45 min in total. They started with a practice phase, consisting of four short blocks of 24 trials in total. Practice blocks 1–3 were single-task blocks where they practiced each task separately; practice block 4 was a mixed-task block where all three tasks were intermixed, and every trial was a task switch. The experimental phase consisted of 4 blocks with 120 trials each in total; the experimental blocks 1 and 2 constituted the baseline phase, experimental blocks 3 and 4 the reward phase^2^. Participants could take a short self-paced break after each block. Every trial was a task switch, and pseudo-random task sequences were created a priori that adhered to the following constraints: There was an almost equal number of ABA (N-2 repetitions) and CBA (N-2 switches) task sequences per block (range 58-60 trials per block), and it was controlled that the trial type in the current trial (N-2 repetition or switch) was independent from the trial type in the preceding trial (i.e., the N-3 effect was controlled, see Schuch & Grange, 2015). Moreover, each task occurred equally often (i.e., 40 times per block), and each possible task-stimulus combination occurred equally often (i.e., 5 times per block). The stimulus presented in trial N could not be the same as the stimulus presented in trial N-1, or trial N-2.

#### Design

For the analysis of global reward effects, we employed a three-factorial design with the within-subjects independent variables task sequence (ABA vs. CBA) and phase (baseline phase vs. reward phase), and the between-subject variable motivation group (reward group vs. control group).^3^ The dependent variables were RT and error rates, as well as the three diffusion-model parameters drift rate, boundary separation, and non-decision time.

### Results I: Global reward effects

#### Number of rewarded trials

As manipulation check, we first analyzed the number of trials in which participants received reward feedback during the reward phase. Participants in the reward group could reach a score between 0 and 240 points in the reward phase (because it consisted of 240 trials). On average, participants reached 163.9 points (range 103–229 points, *SD* = 25.9), corresponding to an average reward rate of 68.3%. Participants performing the digit-categorization tasks reached 161.4 points on average (range 115–203 points, *SD* = 23.0); participants performing the face-categorization tasks reached 166.3 points on average (range 103–229 points, *SD* = 28.2).

We also analyzed control group performance and computed the number of trials that would have been rewarded in the control group according to the criteria applied to the reward group. On average, participants in the control group had 97.9 fast trials (range 34–176, *SD* = 27.4), corresponding to a virtual reward rate of 40.8%. Participants performing the digit-categorization tasks had an average of 87.3 fast trials (range 34–141, *SD* = 24.1), corresponding to a virtual reward rate of 36.4%; participants performing the face-categorization tasks had an average of 108.5 fast trials (range 69–176, *SD* = 26.5), corresponding to a virtual reward rate of 45.2%.

When comparing the reward rate in the reward group and the virtual reward rate in the control group, the reward group reached significantly more (virtual) points than the control group (163.9 points vs. 97.9 virtual points, respectively; *t*(95) = 12.07, *p* < .01), indicating that participants in the reward group adapted their performance to achieve a high number of points.

#### Data filtering and ANOVA design

**Fig. 1:**
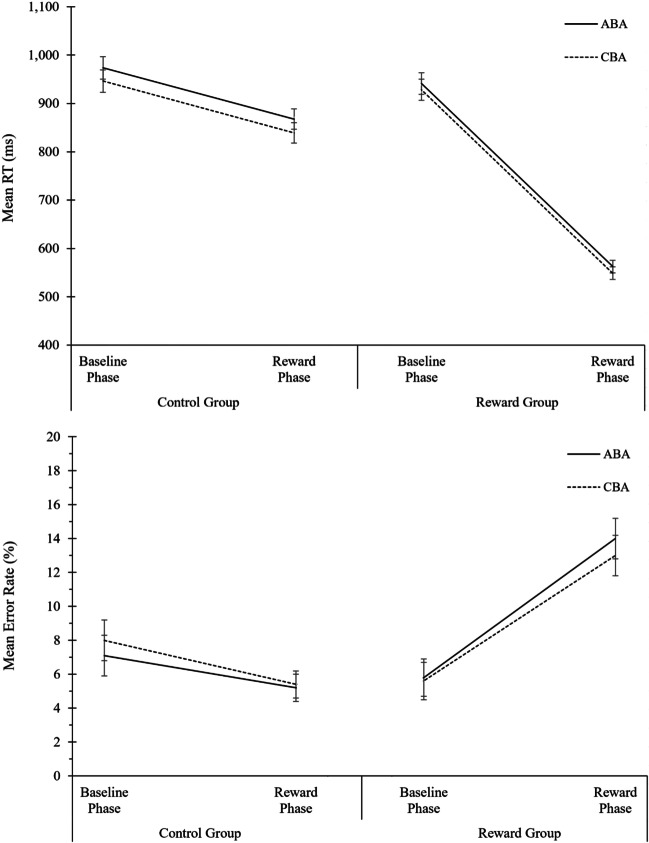

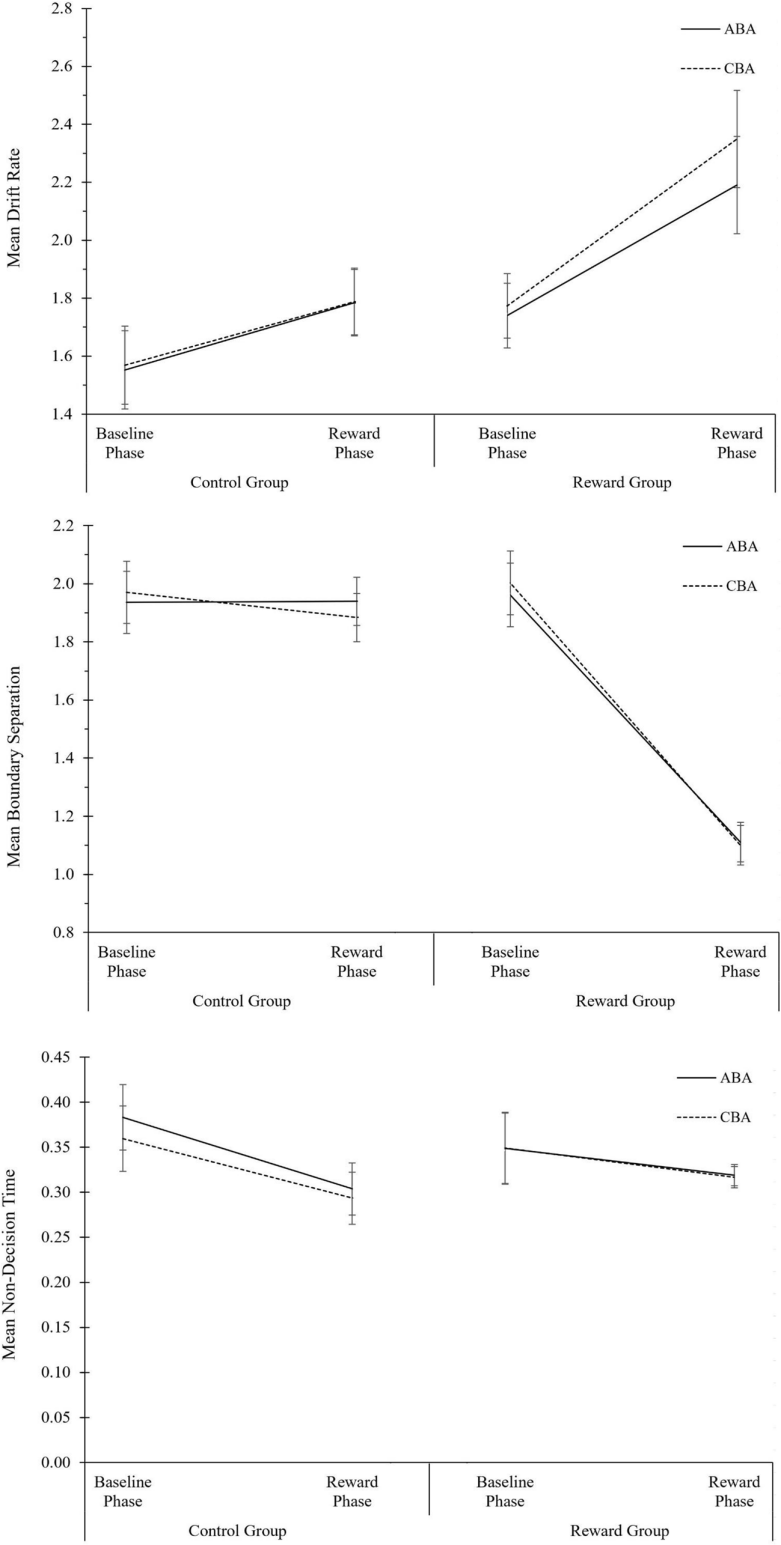
Analysis of global reward effects in Experiment 1. Performance and diffusion model parameters as a function of motivation group (control group, reward group), phase (baseline phase, reward phase), and task sequence (ABA, CBA). From top to bottom: Mean RT, mean error rates, mean drift rate, mean boundary separation, and mean non-decision time. Error bars indicate the 95% confidence interval of the ABA-CBA difference per experimental phase (Pfister & Janczyk, 2013). N = 48 in control group, N = 49 in reward group

Outliers were defined as trials with an RT above or below three standard deviations of the individual participant’s mean per condition and were excluded from analysis (1.7% of trials), as well as the first two trials of each block (1.7%) and the two trials following each error (to exclude error aftereffects, 14.6%). For analysis of RT data, error trials were excluded as well. The mean number of trials per condition and participant included in the analysis of error rates was 98.8 (*SD* = 13.4, range 44–118); for the analysis of RT data, it was 91.7 (*SD* = 17.6, range 26–118). Separate three-way ANOVAs were conducted employing aforementioned design (see Method). The complete ANOVA results are displayed in Table 1. The descriptive data are shown in Fig. 1. Below, we briefly summarize the most important findings.

**Table 1:** Analysis of global reward effects in Experiment 1. Analysis of Variance (ANOVA) on the behavioral measures reaction times (RTs) and error rates, and on the diffusion-model parameters drift rate, boundary separation, and non-decision time

*N* = 49 in Reward Group	RT			Error rate			Drift rate			Boundary separation			Non-decision time		
*N* = 48 in Control Group															
Source of variance	*F*(1,95)	*p* value	${\mathrm n}_p^2$	*F*(1,95)	*p* value	${\mathrm n}_p^2$	*F*(1,95)	*p* value	${\mathrm n}_p^2$	*F*(1,95)	*p* value	${\mathrm n}_p^2$	*F*(1,95)	*p* value	${\mathrm n}_p^2$
Phase	204.79	0.00	0.68	32.40	0.00	0.25	30.55	0.00	0.24	188.76	0.00	0.67	14.64	0.00	0.13
Task Sequence	16.74	0.00	0.15	0.01	0.92	0.00	2.15	0.15	0.02	0.01	0.93	0.00	1.12	0.29	0.01
Motivation Group	6.92	0.01	0.07	9.20	0.00	0.09	5.61	0.02	0.06	14.72	0.00	0.13	0.01	0.95	0.00
Phase * Task Sequence	0.02	0.90	0.00	2.28	0.13	0.02	0.84	0.36	0.01	2.61	0.11	0.03	0.14	0.71	0.00
Phase * Motivation Group	64.53	0.00	0.41	106.62	0.00	0.53	4.62	0.03	0.05	156.20	0.00	0.62	2.35	0.13	0.02
Task Sequence * Motivation Group	2.08	0.15	0.02	4.91	0.03	0.05	1.42	0.24	0.02	0.30	0.59	0.00	0.92	0.34	0.01
Phase * Task Sequence * Motivation Group	0.00	0.99	0.00	0.01	0.95	0.00	1.25	0.27	0.01	0.19	0.66	0.00	0.33	0.57	0.00

#### Reaction times

In RTs, a main effect of task sequence was obtained, *F*(1,95) = 16.74, *p* < .01, η^2^_p_ = .15, indicating N-2 repetition costs of 21 ms. Task sequence did not interact significantly with any of the other factors; that is, we did not observe any modulation of N-2 repetition costs by the reward manipulation (or by the specific paradigm applied, see Open Science Framework (OSF) Additional Material: Part I). Moreover, there were significant main effects of phase, *F*(1,95) = 204.79, *p* < .01, η^2^_p_ = .68, and motivation group, *F*(1,95) = 6.92, *p* = .01, η^2^_p_ = .07,which were further qualified by a two-way interaction, *F*(1,95) = 64.53, *p* < .01, η^2^_p_ = .41. RTs were faster in the reward phase than in the baseline phase, and this effect was larger in the reward group (556 vs. 935 ms) than control group (853 vs. 960 ms).

#### Error rates

In error rates, the main effect of task sequence was not significant. Task sequence did not interact with any of the other factors, except for a significant interaction of task sequence and motivation group, *F*(1,95) = 4.91, *p* < .03, η^2^_p_ = .05, indicating larger N-2 repetition costs in the reward group (0.6%) than in the control group (-0.6%). When tested separately for each group, N-2 repetition costs were not significant in either group; reward group: *t*(48) = 1.48, *p* = .15; control group: *t*(48) = 1.72, *p* = .09. The ANOVA revealed a significant interaction of phase and motivation group, *F*(1,95) = 106.62, *p* < .01, η^2^_p_ = .53, indicating that for the reward group, error rates increased from 5.7% in the baseline phase to 13.5% in the reward phase, whereas in the control group, error rates decreased from the baseline phase (7.5%) to the reward phase (5.3%). There were also significant main effects of phase, *F*(1,95) = 32.04, *p* < .01, η^2^_p_ = .25, and of motivation group, *F*(1,95) = 9.20, *p* < .01, η^2^_p_ = .09.

#### Diffusion modeling of global reward effects

Data filtering was the same as for the analysis of mean error rates as described above, except that outliers for diffusion model analysis were defined according to the procedure recommended by Schmiedek et al. (2007),^4^ excluding trials with RTs faster than 200 ms and trials with RTs larger than four standard deviations above each participant’s mean per experimental condition; this process was repeated on the remaining trials until there were no further outliers (1.1% of the trials were defined as outliers in this way). The mean number of trials per condition and participant included for diffusion modeling was 99.1 (*SD* = 13.8, range 37–118). The software fast-dm (Voss et al., 2015; Voss & Voss, 2007) was used to estimate the parameters drift rate (v), boundary separation (a), and non-decision time (t0), separately for each individual and each condition. To improve model fit, variability of non-decision time (st0) was allowed to vary as well. The starting point bias was set to 0.5*a (i.e., in the middle between the two boundaries); all other parameters implemented in fast-dm were set to 0. The upper and lower boundaries corresponded to correct and error responses, respectively. We used the Kolmogorov-Smirnov (KS) statistic as optimization criterion; the *p* values of the KS statistic did not reveal any significant deviation between observed data and modeled data (*p* values ranged from .18 to 1.00), except for one participant in one condition (where the *p* value was equal to .05). In addition, model fit was inspected graphically by plotting the empirical data against the data predicted by the model (see OSF Additional Material: Part II, Fig. A2); upon visual inspection, all data were included in the analysis.

For statistical analysis, separate ANOVAs were computed on the mean parameter values of boundary separation, drift rate, and non-decision time, employing the design as described above (see *Method*). The complete ANOVA results are displayed in Table 1; the descriptive data are shown in Fig. 1. Below, we summarize the most important findings.

##### Drift rate

There was no significant main effect or interaction of task sequence with any of the other factors. Numerically, the mean drift rate was lower in ABA than CBA trials (1.82 vs. 1.87 evidence units per second as calculated with fast-dm software), but this effect was not statistically significant (*F*(1,95) = 2.15, *p* = .15, η^2^_p_ = .02). Significant main effects of phase, *F*(1,95) = 30.55, *p* < .01, η^2^_p_ = .24, and motivation group were observed, *F*(1,93) = 5.61, *p* = .02, η^2^_p_ = .06, which were further qualified by a two-way interaction, *F*(1,95) = 4.62, *p* = .03, η^2^_p_ = .05. Drift rate was higher in the reward phase than baseline phase (2.03 vs. 1.66), and this increase in drift rate was more pronounced in the reward group than control group (difference of 0.51 vs. 0.23).

##### Boundary separation

No significant main effect or interaction of task sequence with any of the other factors was observed. There were significant main effects of phase, *F*(1,95) = 188.76, *p* < .01, η^2^_p_ = .67, and motivation group, *F*(1,95) = 14.72, *p* < .01, η^2^_p_ = .13, which were further qualified by a two-way interaction, *F*(1,95) = 156.20, *p* < .01, η^2^_p_ = .62. Boundary separation was lower in the reward phase than in the baseline phase (1.51 vs. 1.97 evidence units as calculated with fast-dm software), and this drop in boundary separation was considerably more pronounced in the reward group than in the control group (difference of 0.88 vs. 0.04).

##### Non-decision time

The only significant effect was a main effect of phase, *F*(1,95) = 14.64, *p* < .01, *η*^*2*^_*p*_ = .13, indicating lower non-decision times in the reward phase than in the baseline phase.

### Interim discussion of global reward effects

We observed small overall N-2 repetition costs of 21 ms in RT data, but we did not observe any reward-related modulation of these costs in RT or error rate. Also, while N-2 repetition costs have been observed in the drift rate parameter in previous studies (Schuch, 2016; Schuch & Grange, 2019; Schuch & Konrad, 2017), with a lower drift rate in the last trial of an ABA than CBA sequence, this effect did not reach significance in Experiment 1. Hence, it seems that N-2 repetition costs were small in the present experiment, and this could be a reason for not finding any modulation of these costs by reward, despite a rather large sample (*N* = 97).^5^

At the same time, the reward manipulation produced large effects, with participants in the reward group responding faster and less accurate during the reward phase (see Zhang et al., 2016, for a similar finding). Diffusion modeling revealed a drop in boundary separation, but also an increase in drift rate during the reward phase, especially in the reward group. Implications and underlying mechanisms are addressed in the *General discussion*.

An interesting question is whether this increase in drift rate during the reward phase is mainly triggered by the prospect of reward in the upcoming trial (reward expectancy), or by having experienced a reward in the just-preceding trial (reward experience). In the former case, the increase in drift rate should be present throughout the reward phase, because in the present paradigm participants could potentially receive a reward on every trial during this phase. In the latter case, the increase in drift rate should be modulated by N-1 reward: The increase in drift rate should be larger when the preceding trial had been rewarded than when it had not.

### Results II: Local reward effects

To investigate the question of whether having experienced a reward in the preceding trial modulates performance in the subsequent trial, we performed a second analysis investigating local trial-by-trial effects during the reward phase. To this end, we separated the correct trials of the reward group according to whether the preceding trial N-1 had or had not been rewarded. Notably, such an effect in the reward group could be caused by either the just-experienced reward, or by the good (i.e., fast) performance in the previous trial (because reward was performance-contingent in the present study). In order to distinguish between these two possibilities, we also conducted a trial-by-trial analysis of the reward phase of the control group (where reward did not occur, hence any aftereffects must be due to performance). We separated control group trials according to performance in trial N-1 (hypothetically rewarded or non-rewarded trials had participants been assigned to the reward group) applying the same criteria as in the reward group. Any aftereffects observed in both groups can be attributed to previous-trial performance; any difference in aftereffects between reward group and control group can be attributed to previous-trial reward experienced in the reward group.

#### ANOVA design and data filtering

We employed a three-factorial design with the within-subject independent variables task sequence (ABA vs. CBA) and N-1 reward (trial N-1 rewarded vs. not rewarded), and the between-subject variable motivation group (reward group vs. control group). Data trimming was the same as for the analysis of global reward effects, except that only data from the reward phase were included. Several participants in the reward group had a low trial count in one of the “N-1 not rewarded” conditions. For diffusion modeling with the fast-dm software, a minimum of 10 trials per condition is required; based on this criterion, 13 participants from the reward group had to be excluded. Hence, the analysis of local aftereffects was computed on *N* = 48 participants in the control group and *N* = 36 participants in the reward group. For the analysis of RT data, the mean number of trials per participant and condition was 49.0 (*SD* = 15.2) in the “N-1 rewarded” conditions, and 41.4 (*SD* = 24.7) in the “N-1 not rewarded” conditions. For the analysis of error rates, the mean number of trials per participant and condition was 51.5 (*SD* = 15.2) in the “N-1 rewarded” conditions, and 46.3 (*SD* = 26.8) in the “N-1 not rewarded” conditions. For the analysis of mean performance (but not for diffusion model analysis), we additionally conducted the analyses of local reward aftereffects with all participants included and obtained a similar data pattern (see OSF Additional Material: Part IV, Table A4).

**Fig. 2:**
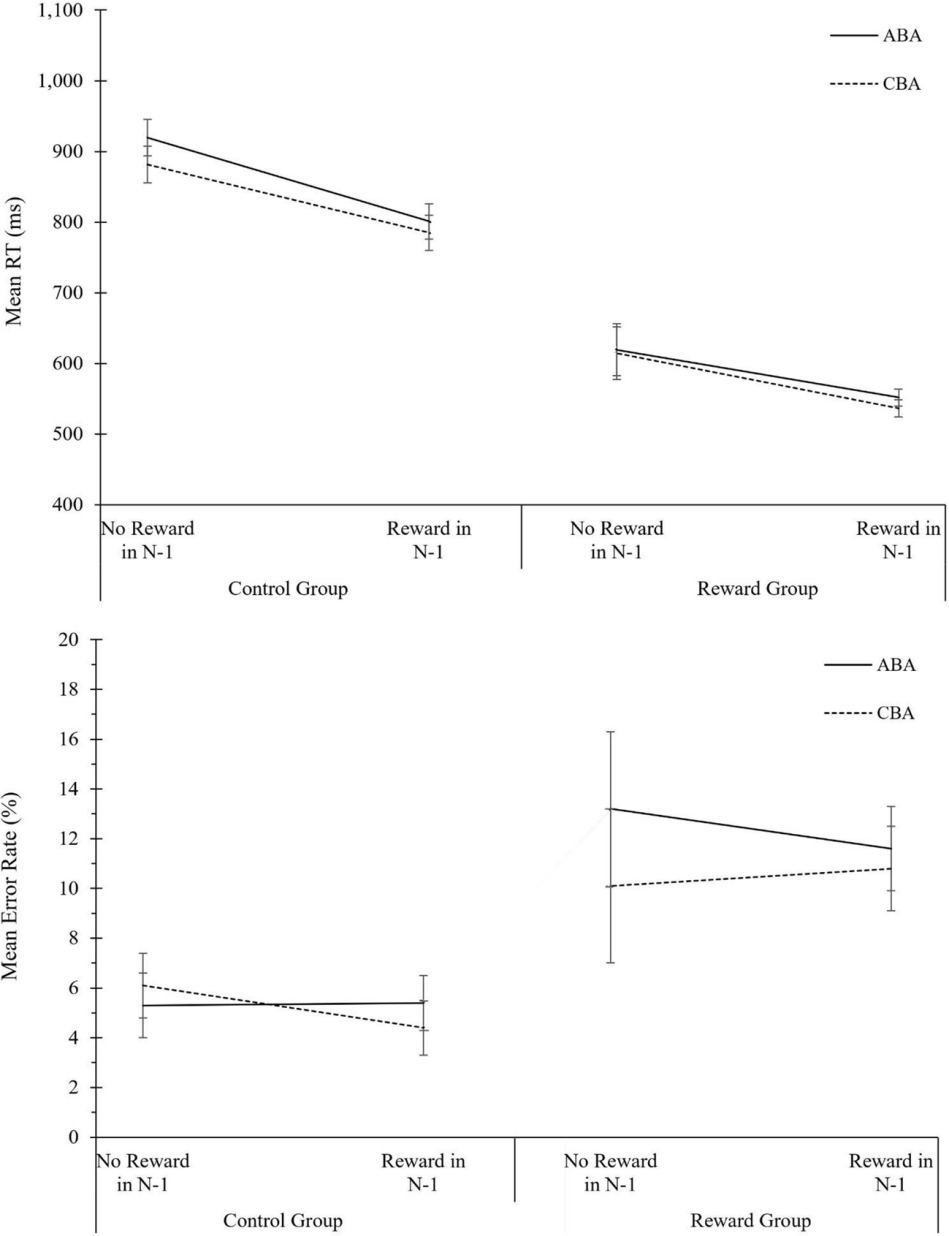

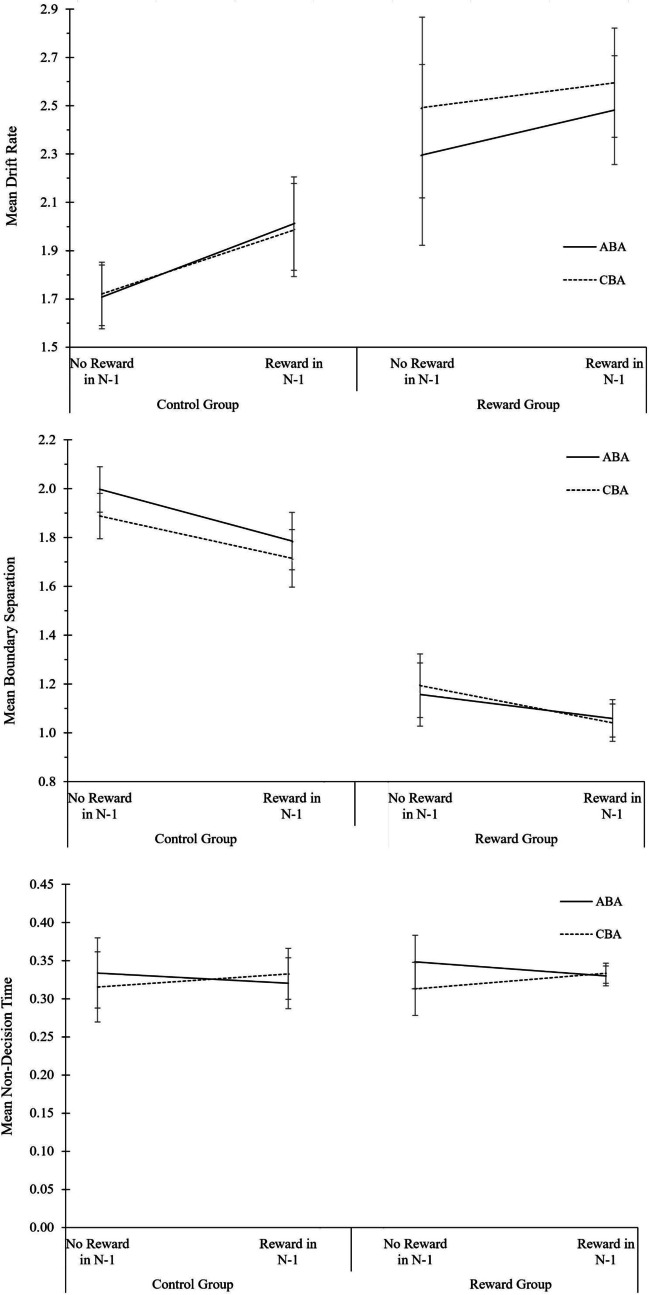
Analysis of local reward aftereffects in the reward phase of Experiment 1. Performance and diffusion model parameters as a function of motivation group (control group, reward group), N-1 reward (not rewarded, rewarded), and task sequence (ABA, CBA). From top to bottom: Mean RT, mean error rates, mean drift rate, mean boundary separation, and mean non-decision time. Error bars indicate the 95% confidence interval of the ABA-CBA difference per experimental phase (Pfister & Janczyk, 2013). N = 48 in control group, N = 36 in reward group

The descriptive data are plotted in Fig. 2; the complete ANOVA results are shown in Table 2. Regarding diffusion model fit, the *p* values of the KS statistic did not reveal any significant deviation between observed data and modeled data (*p* values ranging from .32 to 1.00; see OSF Additional Material: Part II, Fig. A3 for a visualization of diffusion model fit). Here, we summarize the most important results of the local reward effects analysis.

**Table 2:** Analysis of local reward effects in Experiment 1. Analysis of Variance (ANOVA) on the behavioral measures reaction times (RTs) and error rates, and on the diffusion-model parameters drift rate, boundary separation, and non-decision time

*N* = 36 in Reward Group	RT			Error rate			Drift rate			Boundary separation			Non-decision time		
*N* = 48 in Control Group															
Source of variance	*F*(1,82)	*p* value	${\mathrm n}_p^2$	*F*(1,82)	*p* value	${\mathrm n}_p^2$	*F*(1,82)	*p* value	${\mathrm n}_p^2$	*F*(1,82)	*p* value	${\mathrm n}_p^2$	*F*(1,82)	*p* value	${\mathrm n}_p^2$
N-1 Reward	66.17	0.00	0.45	1.37	0.25	0.02	9.08	0.00	0.10	33.14	0.00	0.29	0.03	0.85	0.00
Task Sequence	6.07	0.02	0.07	5.87	0.02	0.07	1.67	0.20	0.02	2.29	0.13	0.03	1.21	0.28	0.02
Motivation Group	16.35	0.00	0.17	36.28	0.00	0.31	11.01	0.00	0.12	60.30	0.00	0.42	0.84	0.00	0.84
N-1 Reward * Task Sequence	0.25	0.62	0.00	0.05	0.82	0.00	0.27	0.61	0.00	0.02	0.89	0.00	0.08	0.04	0.08
N-1 Reward * Motivation Group	2.50	0.12	0.03	0.15	0.70	0.00	0.97	0.33	0.01	1.52	0.22	0.02	0.00	0.96	0.00
Task Sequence * Motivation Group	1.22	0.27	0.02	4.96	0.03	0.06	2.01	0.16	0.02	3.48	0.07	0.04	0.57	0.45	0.01
N-1 Reward * Task Sequence * Motivation Group	1.92	0.17	0.02	4.11	0.05	0.05	0.03	0.85	0.00	0.70	0.40	0.01	0.82	0.00	0.82

*Note.* Thirteen participants in the reward group were excluded from primary analyses due to low trial numbers for diffusion modeling. As crosscheck, analyses of RT and error rate were additionally conducted with all participants (see OSF Additional Material: Part IV, Table A4)

#### Reaction times

Only main effects were found in RT: A main effect of task sequence, *F*(1,82) = 6.07, *p* = .02, η^2^_p_ = .07, indicating N-2 repetition costs, a main effect of motivation group, *F*(1,82) = 16.35, *p* < .01, η^2^_p_ = .17, indicating faster RTs in the reward than control group, and a main effect of N-1 reward, *F*(1,82) = 66.17, *p* < .01, η^2^_p_ = .45, indicating faster performance after fast N-1 trials (that were rewarded in the reward group, but not in the control group) than after slow N-1 trials. N-1 reward did not significantly interact with any of the other factors.

#### Error rates

The three-way ANOVA on error rates revealed a main effect of task sequence, *F*(1,82) = 5.87, *p* < .02, η^2^_p_ = .07, indicating N-2 repetition costs, and a main effect of motivation group, *F*(1,82) = 36.28, *p* < .01, η^2^_p_ = .31, indicating higher error rates in the reward than control group. Moreover, there was an interaction of task sequence and motivation group, *F*(1,82) = 4.96, *p* < .03, η^2^_p_ = .06, indicating larger N-2 repetition costs in the reward group (1.9%) than control group (0.1%). There was also a just-significant three-way interaction of task sequence, motivation group, and N-1 reward, *F*(1,82) = 4.11, *p* < .05, η^2^_p_ = .05. On a descriptive level, N-2 repetition costs in the reward group were larger after non-rewarded trials (3.0%) than after rewarded trials (0.8%), whereas N-2 repetition costs in the control group were smaller after virtually non-rewarded trials (-0.8%) than after virtually rewarded trials (1.0%). However, when analyzing the two groups separately in follow-up ANOVAs with the factors task sequence and N-1 reward, the interaction was not significant in the reward group, *F*(1,82) = 1.38, *p* = .25, η^2^_p_ = .04, and neither in the control group, *F*(1,82) = 3.49, *p* = .07, η^2^_p_ = .07. Moreover, when all participants were included in the analysis, the two-way interaction of task sequence and motivation group, and the three-way interaction with N-1 reward, were no longer significant (see OSF Additional Material: Part IV, Table A4).

#### Diffusion modeling of local reward effects

For the drift rate parameter, a significant main effect of N-1 reward was observed, *F*(1,82) = 9.08, *p* < .01, η^2^_p_ = .10, indicating higher drift rate after fast trials (that were rewarded in the reward group) than after slow trials. N-1 reward did not interact with any of the other factors. A main effect of motivation group was also observed, with higher drift rate in the reward group than control group, *F*(1,82) = 11.01, *p* < .01, η^2^_p_ = .12.

For the boundary separation parameter, a significant main effect of N-1 reward was observed, *F*(1,82) = 33.14, *p* < .01, η^2^_p_ = .29, indicating lower boundary separation after fast trials than after slow trials. A large main effect of motivation group was also observed, with lower boundary separation in the reward group than control group, *F*(1,82) = 60.30, *p* < .01, η^2^_p_ = .42. For the non-decision time parameter, the ANOVA did not yield any significant effects.

### Interim discussion of local reward effects

The analysis of local reward aftereffects revealed faster RTs, as well as higher drift rate and lower boundary separation, after fast N-1 trials (that were rewarded in the reward group, but not in the control group) than after slow N-1 trials. We did not observe any differences in aftereffects between reward and control group, except for a just-significant three-way interaction in error rates, which was no longer significant when all participants were included (an opposite pattern was found in Experiment 2; hence we do not consider this a reliable effect). In the *General discussion* we elaborate on why the observed effects are attributable on reward expectancy as opposed to acutal reward experience.

Why do we observe faster RTs after fast N-1 trials than after slow N-1 trials in both groups? One possibility is that performance fluctuates over the course of the experiment, with phases of good performance (lasting for several trials) alternating with phases of less good performance (again lasting for several trials). Hence, fast trials would tend to be followed by another fast trial, and slow trials by another slow trial. In a similar vein, errors are usually not evenly spread over the duration of an experiment, but tend to occur in bundles (Dutilh et al., 2012), suggesting that performance fluctuates over the course of an experiment. Such fluctuations of performance could be due to “lapses of attention,” as they have been reported in the literature on “mind wandering” (for reviews, see, e.g., Handy & Kam, 2015, Mooneyham & Schooler, 2013; Seli et al., 2016; Smallwood & Schooler, 2015; see also Esterman et al., 2017, for neuroimaging evidence of attentional fluctuations and their modulation by reward). In the present data, we observed that the faster RTs after fast (vs. slow) N-1 trials were associated with a higher drift rate. This difference in drift rate is in line with the idea of attentional fluctuations, with higher drift rate corresponding to an attentional state that is more focused on the current task. We also observed a lower diffusion model boundary separation after fast (vs. slow) N-1 trials. This could indicate that participants’ response strategy also fluctuates over the course of the experiment, with more cautious responding after slow trials, where attention is less focused on the current task.

## Experiment 2

Addressing the lack of a reward-based modulation of N-2 repetition costs, we conceptually replicated Experiment 1 with another version of the N-2 task switching paradigm and a slightly modified reward manipulation. In particular, in Experiment 2, we used pictures of household items as stimuli, all of which were emotionally neutral. Participants switched between three different categorization tasks of the household items. Further, we modified our reward manipulation to include penalties for incorrect responses, in order to foster the requirement for inhibitory control and to prevent an overemphasis on speed over accuracy.

### Method

#### Participants

The sample comprised 96 new participants, which were again students or friends of students from RWTH Aachen University. Half of them were randomly assigned to the reward group (36 female, 11 male, one diverse; age: *M* = 22.6 years, *SD* = 4.0 years, range 18–39 years), and the other half to the control group (35 female, 13 male, age: *M* = 23.5 years, *SD* = 4.4 years, range 18–34 years). The acquisition and compensation of participants proceeded in the same way as in Experiment 1.

#### Task, stimuli, and responses

Forty different pictures of ubiquitous household items were used as stimuli. Each picture showed a different household item, measured 17 × 19.5 cm, and was taken from the Bank of Standardized Stimuli (Brodeur et al., 2010). Relevant stimulus dimensions were the size, typical storage location, and orientation of the household items, yielding three tasks: Participants had to categorize household items as smaller or larger than a shoebox (size task); being typically located in the kitchen or in the garage (location task); or being displayed upright or upside down (orientation task). Colored frames were used as task cues. A red frame indicated the size task, a blue frame the location task, and a yellow frame the orientation task in the upcoming trial. Response keys, response mappings, and the trial procedure were corresponding to Experiment 1.

#### Reward manipulation

The reward manipulation from Experiment 1 was slightly modified to reduce the focus on speed over accuracy in the reward phase. In case of an incorrect response, participants in the reward group now lost a reward point and received the feedback “Error! – 1 point” (all feedback messages were presented in German). As in Experiment 1, they received the feedback “Correct! + 1 point” for correct and fast responses, and “Slow! No point” for correct but too slow responses. Participants in the control group received the feedback message “Error!” after incorrect responses, “Correct!” after correct and fast responses, and “Slow!” after correct and slow responses. The performance-dependent feedback message “Slow!” was newly introduced in order to make the feedback messages in the reward phase as similar as possible between the two groups.

#### Procedure and design

Two additional reward blocks with 120 trials each were added in Experiment 2 in order to increase trial numbers. Thus, Experiment 2 comprised four practice blocks à 24 trials, two baseline blocks à 120 trials, and four reward blocks à 120 trials. The analysis design was identical to Experiment 1.

### Results I: Global reward effects

#### Number of rewarded trials

A maximum of 480 (virtual) points could be scored during the four reward blocks in Experiment 2. Reward scores were significantly higher in the reward group (*M* = 287.0 points, *SD* = 88.2, range 5–429; corresponding to 59.8% of the maximally attainable score) than virtual reward scores in the control group (*M* = 254.1 virtual points, *SD* = 86.6, range -6–367; 52.9% of the maximally attainable virtual score), *t*(94) = 1.85, *p* = .034, one-tailed.

#### Data filtering

Data filtering was conducted as in the analysis of global reward effects in Experiment 1. Practice blocks, the first two trials of each block (1.7%), the first two post-error trials (19.1%), and outliers (1.8%) were excluded. For RT analysis, error trials (9.5%) were also removed. For diffusion model analysis, outliers were defined following Schmiedek et al. (2007) and discarded (1.5%). On average, 141.2 trials (range 100.6–181.9 trials) remained for each subject and condition for diffusion modeling. *P*-values from the KS statistics indicating model fit ranged from .13 to 1.00. Graphical inspection of empirical data plotted against data predicted by the model indicated poor model fit for four participants in the control group, which were therefore excluded from all analyses. For the remaining participants (44 in control group, 48 in reward group), diffusion model fit was good (see OSF Additional Material: Part II, Fig. A4). For completeness, we also report the analyses of Experiment 2 with all participants included in the OSF Additional Material: Part IV, Table A5; the data pattern was similar.

#### Data analysis

**Fig. 3:**
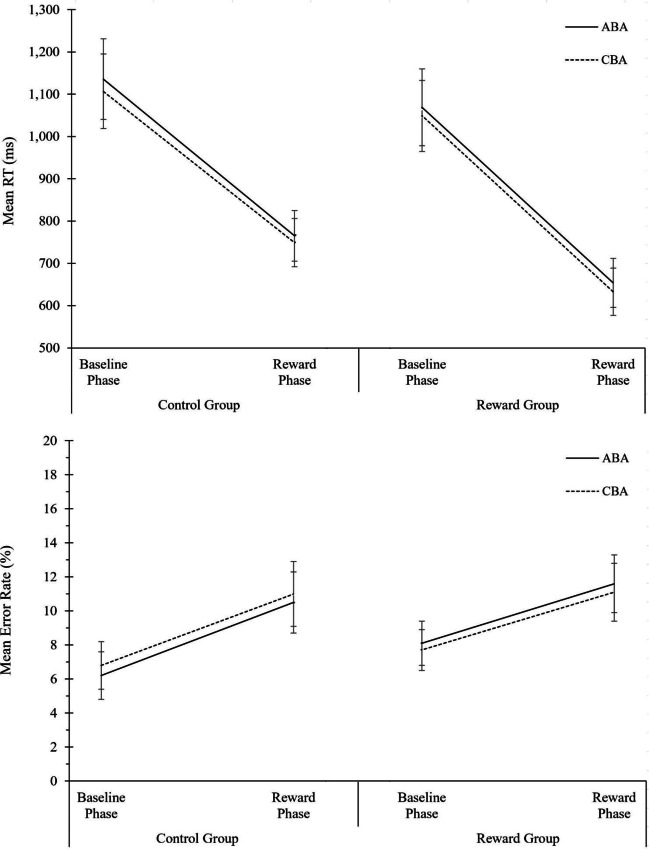

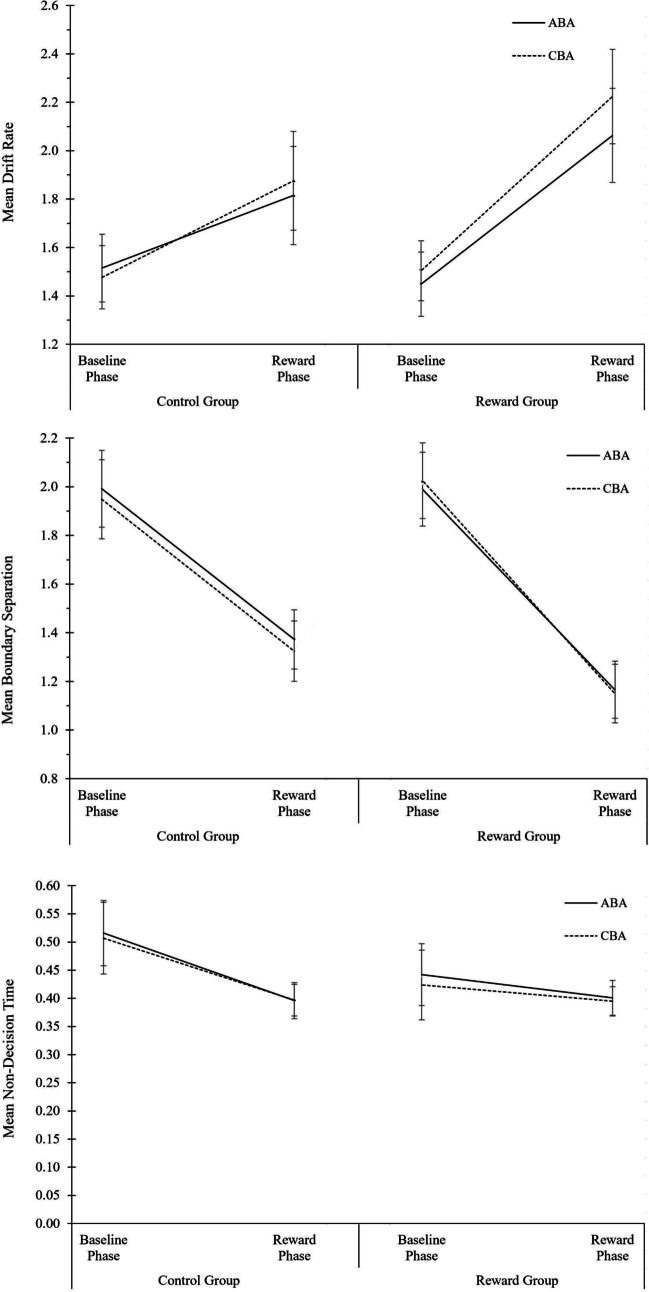
Analysis of global reward effects in Experiment 2. Performance and diffusion model parameters as a function of motivation group (control group, reward group), phase (baseline phase, reward phase), and task sequence (ABA, CBA). From top to bottom: Mean RT, mean error rates, mean drift rate, mean boundary separation, and mean non-decision time. Error bars indicate the 95% confidence interval of the ABA-CBA difference per experimental phase (Pfister & Janczyk, 2013). N = 44 in control group, N = 48 in reward group

Data analysis procedure was identical to the analysis of global reward effects in Experiment 1. The complete ANOVA results for behavioral data and diffusion model parameters are displayed in Table 3. The descriptive data are shown in Fig. 3.

**Table 3:** Analysis of global reward effects in Experiment 2. Analysis of Variance (ANOVA) on the behavioral measures reaction times (RTs) and error rates, and on the diffusion-model parameters drift rate, boundary separation, and non-decision time

*N* = 48 in Reward Group	RT			Error rate			Drift rate			Boundary separation			Non-decision time		
*N* = 44 in Control Group															
Source of variance	*F*(1,90)	*p* value	${\mathrm n}_p^2$	*F*(1,90)	*p* value	${\mathrm n}_p^2$	*F*(1,90)	*p* value	${\mathrm n}_p^2$	*F*(1,90)	*p* value	${\mathrm n}_p^2$	*F*(1,90)	*p* value	${\mathrm n}_p^2$
Phase	354.03	0.00	0.80	73.54	0.00	0.45	95.20	0.00	0.51	315.86	0.00	0.78	19.69	0.00	0.18
Task Sequence	22.10	0.00	0.20	0.06	0.81	0.00	4.35	0.04	0.05	0.75	0.39	0.01	0.99	0.32	0.01
Motivation Group	3.23	0.08	0.04	1.21	0.28	0.01	1.88	0.17	0.02	0.81	0.37	0.01	2.11	0.15	0.02
Phase * Task Sequence	0.34	0.56	0.00	0.05	0.82	0.00	4.47	0.04	0.05	0.45	0.51	0.01	0.58	0.45	0.01
Phase * Motivation Group	1.53	0.22	0.02	0.83	0.37	0.01	9.27	0.00	0.09	7.61	0.01	0.08	5.68	0.02	0.06
Task Sequence * Motivation Group	0.04	0.85	0.00	3.00	0.09	0.03	2.86	0.09	0.03	1.69	0.20	0.02	0.23	0.64	0.00
Phase * Task Sequence * Motivation Group	0.60	0.44	0.01	0.00	0.98	0.00	0.01	0.93	0.00	0.28	0.60	0.00	0.02	0.90	0.00

#### Reaction times

Significant main effects of phase, *F*(1, 90) = 354.03, *p <* .001, ${\mathrm n}_p^2$ = .80, and task sequence, *F*(1, 90) = 22.10, *p* = .001, ${\mathrm n}_p^2$ = .20, were found. Responses were faster in the reward phase compared to the baseline phase (700 vs. 1,090 ms), and slower in ABA than in CBA task sequences (906 vs. 885 ms) yielding N-2 task repetition costs of 21 ms. On a descriptive level, N*-2* task repetition costs were 24 ms in the baseline phase, and 18 ms in the reward phase, however, the interaction of phase and task sequence was not significant, *F*(1, 90) < 1.

#### Error rates

The main effect of phase was significant, *F*(1, 90) = 73.54, *p* < .001, ${\mathrm n}_p^2$ = .45, indicating that more errors occurred in the reward phase than in the baseline phase (11.9 vs. 7.2%).

#### Diffusion modeling of global reward effects

For drift rate, the main effect of phase, *F*(1, 90) = 95.20, *p* < .001, ${\mathrm n}_p^2$ = .51, as well as the interaction between motivation group and phase, *F*(1, 90) = 9.27, *p* = .003, ${\mathrm n}_p^2$ = .09, were significant. Drift rate was higher in the reward phase than baseline phase (1.99 vs. 1.49), while this increase was more pronounced in the reward group than control group (difference of 0.67 vs. 0.35). Moreover, the main effect of task sequence, *F*(1, 90) = 4.35, *p* = .040, ${\mathrm n}_p^2$ = .05, and the two-way interaction of phase and task sequence, *F*(1, 90) = 4.47, *p* = .037, ${\mathrm n}_p^2$ = .05, reached significance. Drift rate was lower in in the last trial of an ABA than CBA task sequence (1.71 vs. 1.77), and these N-2 task repetition costs were higher in the reward phase than in the baseline phase (0.11 vs. 0.01).

For boundary separation, the main effect of phase, *F*(1, 90) = 315.86, *p* < .001, ${\mathrm n}_p^2$ = .78, and the interaction of phase and motivation group, *F*(1, 90) = 7.61, *p* = .007, ${\mathrm n}_p^2$ = .08, were significant. Boundary separation decreased strongly from the baseline phase to the reward phase (1.99 vs. 1.25), and this decrease was more pronounced in the reward group than in the control group (difference of 0.85 vs. 0.62).

Regarding non-decision time, a significant main effect of phase, *F*(1, 90) = 19.69, *p* < .001, ${\mathrm n}_p^2$ = .18, and a significant interaction of motivation group and phase, *F*(1, 90) = 5.68, *p* = .019, ${\mathrm n}_p^2$ = .06, indicated a lower non-decision time in the reward phase relative to the baseline phase, and the decrease in non-decision time was less pronounced in the reward group than in the control group.

### Interim discussion of global reward effects

Significant differences between the average reward rate in the reward group and the virtual reward rate in the control group indicate a successful reward manipulation, although the between-group difference was numerically smaller in Experiment 2 than in Experiment 1 (8% vs. 28%; see *General discussion* for more details). The ANOVA results largely coincide with the results of Experiment 1: There was an increase in response speed and decrease in boundary separation from baseline phase to reward phase. The latter was more pronounced in the reward group than in the control group, likely reflecting a shift in response strategy from the baseline phase to the reward phase induced by the reward manipulation. There were also some minor differences between the data patterns in Experiments 1 and 2: In Experiment 2, the reward group and control group did not differ statistically with respect to overall accuracy level and boundary separation, and in RT data, the interaction between phase and motivation group was no longer significant. These differences between experiments are likely due to the slight changes in reward schedule between Experiments 1 and 2.

Importantly, in Experiment 2 as well as in Experiment 1, the increase in drift rate from baseline phase to reward phase was larger in the reward group than control group. See *General discussion* for more details on why this result shows that the reward manipulation did not just induce a speed-accuracy tradeoff, and for considerations that reward expectancy may sharpen task set representations (Yee & Braver, 2018) and/or increase visual attention (Grahek et al., 2021).

Moreover, in Experiment 2, there was a significant interaction of phase and task sequence in drift rate across both groups, suggesting that the increased difficulty in evidence accumulation in ABA relative to CBA task sequences was more pronounced in the reward phase (but this effect was not specific to the reward group). This data pattern is different to Experiment 1, where we had observed a significant interaction of phase and motivation group in drift rate (but this effect was not specific to ABA vs. CBA task sequences). No other modulations of N-2 task repetition costs were observed in the analysis of global reward effects in either Experiment 1 or 2.

### Results II: Local reward effects

As in Experiment 1, we performed a second analysis zooming into a trial-by-trial level in the reward phase to investigate whether N-2 task repetition costs are modulated by reward locally. Performance differences between reward group and control group subsequent to a fast trial would imply effects due to reward experience. A modulation of N-2 task repetition costs after fast correct trials in the reward group would suggest a local reward-based enhancement of task-set inhibition.

#### Data filtering

The four control-group participants that were excluded from the global analysis (due to poor diffusion model fit) were excluded from the local analysis as well. As with Experiment 1, data trimming for the local analysis was the same as for the global analysis, except that only data from the reward phase were included. Three participants in the reward group and one further participant in the control group had less than 10 trials in one of the “N-1 not rewarded” conditions and therefore were also excluded from the local analysis (as in Experiment 1). Thus, in total, 43 participants in the control group and 45 participants in the reward group were included in the analysis of local reward effects.

As for Experiment 1, outliers for diffusion-model analysis were defined according to Schmiedek et al. (2007) and removed (1.4%). On average, 97.6 trials (range 42.1–152.8 trials) remained for each subject and condition for diffusion model analysis. *P*-values from the KS statistics ranged from .17 to 1.00. Diffusion model fit for the included participants (43 in control group, 45 in reward group) was good (see OSF Additional Material: Part II, Fig. A5 for a graphical illustration of diffusion model fit).

#### Data analysis

**Fig. 4:**
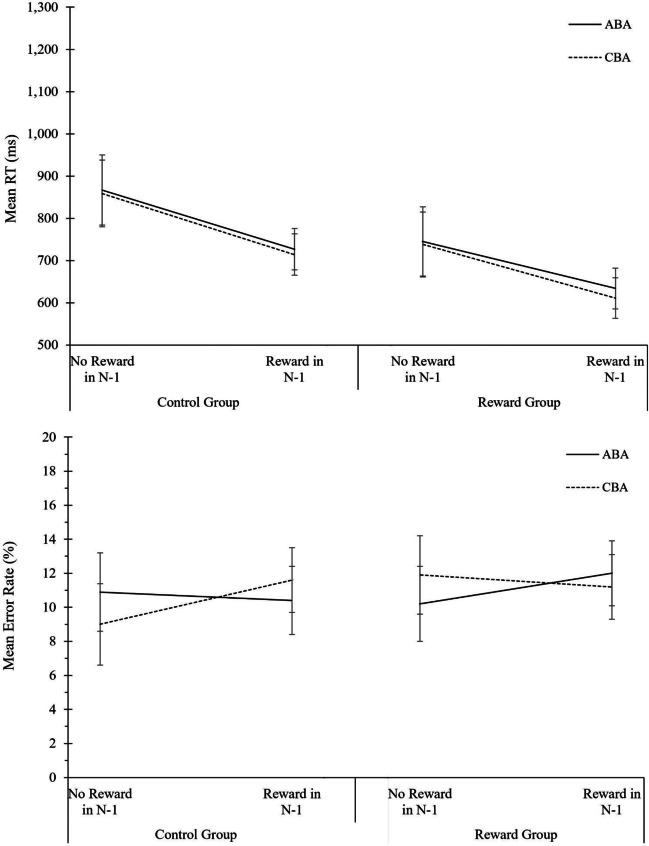

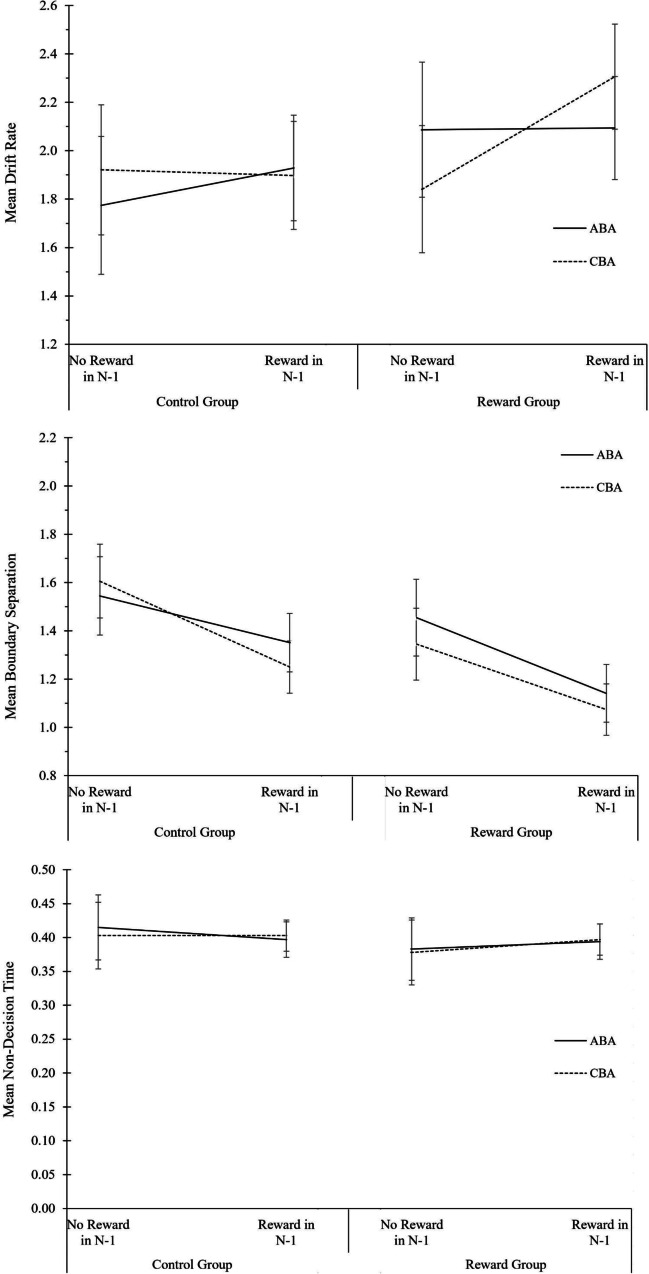
Analysis of local reward aftereffects in the reward phase of Experiment 2. Performance and diffusion model parameters as a function of motivation group (control group, reward group), N-1 reward (not rewarded, rewarded), and task sequence (ABA, CBA). From top to bottom: Mean RT, mean error rates, mean drift rate, mean boundary separation, and mean non-decision time. Error bars indicate the 95% confidence interval of the ABA-CBA difference per experimental phase (Pfister & Janczyk, 2013). N = 43 in control group, N = 45 in reward group

Data analysis was identical to the analysis of local reward effects in Experiment 1. The complete ANOVA results for behavioral data and diffusion model parameters are displayed in Table 4. The descriptive data are shown in Fig. 4. As crosscheck, RT and error analyses were additionally run including all 96 participants (OSF Additional Material: Part IV, Table A6). We further report analyses including participants with poor diffusion model fit but excluding participants with low trial numbers (OSF Additional Material: Part IV, Table A7); the results pattern was similar.

**Table 4:** Analysis of local reward effects in Experiment 2. Analysis of Variance (ANOVA) on the behavioral measures reaction times (RTs) and error rates, and on the diffusion-model parameters drift rate, boundary separation, and non-decision time

*N* = 45 in Reward Group	RT			Error rate			Drift rate			Boundary separation			Non-decision time		
*N* = 43 in Control Group															
Source of variance	*F*(1,86)	*p* value	${\mathrm n}_p^2$	*F*(1,86)	*p* value	${\mathrm n}_p^2$	*F*(1,86)	*p* value	${\mathrm n}_p^2$	*F*(1,86)	*p* value	${\mathrm n}_p^2$	*F*(1,86)	*p* value	${\mathrm n}_p^2$
N-1 Reward	80.74	0.00	0.48	2.77	0.10	0.03	7.81	0.01	0.08	74.32	0.00	0.46	0.05	0.82	0.00
Task Sequence	4.29	0.04	0.05	0.02	0.88	0.00	0.13	0.72	0.00	3.68	0.06	0.04	0.11	0.74	0.00
Motivation Group	6.22	0.02	0.07	0.51	0.48	0.01	1.92	0.17	0.02	5.04	0.03	0.06	0.57	0.45	0.01
N-1 Reward * Task Sequence	0.87	0.35	0.01	0.11	0.75	0.00	1.36	0.25	0.02	1.16	0.29	0.01	0.92	0.34	0.01
N-1 Reward * Motivation Group	0.65	0.42	0.01	0.26	0.61	0.00	2.49	0.12	0.03	0.07	0.80	0.00	0.82	0.37	0.01
Task Sequence * Motivation Group	0.10	0.75	0.00	0.47	0.50	0.01	0.42	0.52	0.01	1.47	0.23	0.02	0.03	0.87	0.00
N-1 Reward * Task Sequence * Motivation Group	0.23	0.63	0.00	8.88	0.00	0.09	7.02	0.01	0.08	3.41	0.07	0.04	0.14	0.71	0.00

*Note.* Five participants in the control group and three participants in the reward group were excluded from primary analyses due to poor diffusion model fits and/or low trial numbers for diffusion modeling. As crosscheck, analyses were additionally conducted with different data filtering criteria (see OSF Additional Material: Part IV, Tables A6 and A7)

#### Reaction times

The main effects of motivation group, *F*(1, 86) = 6.22, *p* = .015, ${\mathrm n}_p^2$ = .07, N-1 reward, *F*(1, 86) = 80.74, *p* < .001, ${\mathrm n}_p^2$ = .48, and task sequence, *F*(1, 86) = 4.29, *p* = .041, ${\mathrm n}_p^2$ = .05, were significant. Participants in the reward group responded faster than participants in the control group (682 vs. 792 ms), and responses were faster after fast N-1 trials (which were rewarded in the reward group) than after slow N-1 trials (671 vs. 802 ms). N-2 task repetition costs amounted to 12 ms.

#### Error rates

Only the three-way interaction of motivation group, N-1 reward, and task sequence was significant, *F*(1, 86) = 8.88, *p* = .004, ${\mathrm n}_p^2$ = .09. N-2 task repetition costs were modulated less by fast N-1 trials in the reward group (2.5%) than in the control group (–3.1%). Descriptively, the reward group showed an N-2 task repetition *benefit* after slow (non-rewarded) N-1 trials (1.7%), and N-2 task repetition costs after fast (rewarded) N-1 trials (0.8%), while the control group showed the opposite pattern, that is, N-2 task repetition costs after slow N-1 trials (1.9%) and an N-2 task repetition benefit after fast N-1 trials (1.2%). When analyzing the two groups separately in follow-up ANOVAs with the factors task sequence and N-1 reward, the interaction remained significant only in the control group, *F*(1,42) = 6.00, *p* = .019, η^2^_p_ = .13.

#### Diffusion modeling of local reward effects

For drift rate, a significant main effect for N-1 reward was observed, *F*(1, 86) = 7.81, *p* = .006, ${\mathrm n}_p^2$ = .08, revealing drift rate was higher in trials following a fast N-1 trial relative to a slow N-1 trial (2.06 vs. 1.91). The three-way interaction of motivation group, N-1 reward, and task sequence was significant, *F*(1, 86) = 7.02, *p* = .010, ${\mathrm n}_p^2$ = .08. N-2 task repetition costs increased after fast N-1 trials relative to slow N-1 trials in the reward group (0.21 vs. – 0.25) but decreased after fast N-1 trials relative to slow N-1 trials in the control group (– 0.03 vs. 0.15). Follow-up ANOVAs conducted separately for the two groups with the factors N-1 reward and task sequence revealed that the main effect of N-1 reward as well as the interaction of N-1 reward and task sequence remained significant only in the reward group, *F*(1, 44) = 8.74, *p* = .005, ${\mathrm n}_p^2$ = .17, and *F*(1, 44) = 8.15, *p* = .007, ${\mathrm n}_p^2$ = .16, respectively.

For boundary separation, the main effects of motivation group, *F*(1, 86) = 5.04, *p* = .027, ${\mathrm n}_p^2$ = .06, and N-1 reward, *F*(1, 86) = 74.32, *p* < .001, ${\mathrm n}_p^2$ = .46, were significant, showing boundary separation was lower in the reward group than in the control group (1.25 vs. 1.44), and after fast N-1 trials relative to slow N-1 trials (1.20 vs. 1.49). No effects were significant for non-decision time, *F*(1, 86) < 1.

### Interim discussion of local reward effects

Converging with the previous results from Experiment 1, RT was faster, drift rate higher, and boundary separation lower in the reward group than in the control group, and after fast trials relative to after slow trials. This pattern confirms both long-term and short-term adjustments in response strategy towards faster responses to maximize reward in the reward group, and to maximize positive feedback in the control group.

The three-way interaction of N-1 reward, task sequence, and motivation group was significant in error rate in Experiment 2. However, the pattern was opposite to the one observed in Experiment 1, and it was no longer significant in the reward group in post-hoc analyses, suggesting the effect was not reliable. The same interaction became significant in drift rate showing that N-2 task repetition costs increased after fast (i.e., rewarded) trials in the reward group. So far, this is the only evidence suggesting a reward-based increase of task-set inhibition, while results in the other dependent variables did not suggest any reward-based modulation thereof.

### Combined analysis of data from both experiments

As we did not find clear evidence concerning a reward-based modulation of N-2 task repetition costs, we combined the data from Experiments 1 and 2 and performed a global and a local analysis thereof analogously to the previously described analyses. With a total sample size of 193 participants, a power analysis with MorePower v6.0.4 (Campbell & Thompson, 2012) revealed a power of .93 to detect a medium-sized (${\mathrm n}_p^2$ = .06) three-way interaction of motivation group, phase, and task sequence, and a power of .80 to detect a small- to medium-sized (${\mathrm n}_p^2$ = .04) three-way interaction. Additionally, we performed Bayesian ANOVAs with JASP software version 0.16.3 (https://jasp-stats.org) on the dependent variables RT and error rate, and on the diffusion model parameters, for global and local reward effects, by comparing the model including the critical three-way interaction of interest to all models excluding the critical three-way interaction (see OSF Additional Material: Part V for a full description of the Method and Results of the Combined Analysis).

The result pattern largely mirrored the effects previously found in Experiments 1 and 2 and did not indicate any reward-based modulation of task-set inhibition. All Bayesian analyses yielded an Exclusion Bayes Factor greater than 3, which according to convention can be interpreted as “substantial evidence” for the absence of the effect of interest (Jeffreys, 1961). Thus, Bayesian analyses implied that the present data is more likely to be found in models *excluding* the critical three-way interaction of phase, motivation group, and task sequence for global reward effects, and *excluding* the three-way interaction of N-1 reward, motivation group, and task sequence for local reward effects.

Overall, task-set inhibition as measured by N-2 task repetition costs appear to be modulated neither by reward expectancy (globally) nor by reward experience (locally) with the reward manipulations employed in Experiments 1 and 2. Note that this combined analysis is somewhat limited since it averages data from two slightly different paradigms (e.g., errors were not penalized in Experiment 1, but in Experiment 2).

N-2 task repetition costs were relatively small (12 ms) in the local analysis, i.e., in the reward phase. One possibility is that effects of two or more fast previous trials (consecutive rewards for the reward group) may have opposing effects on the level of task sets. However, an explorative analysis considering additionally the performance in trial N-2 in our data did not find robust effects to confirm possibly opposing effects of consecutive rewards on the level of task sets (see OSF Additional Material: Part VI).

## General discussion

The present study contributes to research on the interface between motivation and cognitive control. We let participants perform a task switching paradigm that allowed us to measure N-2 repetition costs, which can be taken as a marker of inhibitory control on the task level. We compared performance between two groups: A reward group that received performance-contingent reward in the second part of the experiment (reward phase), and a control group that did not receive any reward. The baseline phase was identical for both groups (no reward could be obtained). Two experiments were conducted that differed slightly with respect to the reward scheme and the task switching paradigm. Regarding reward scheme, in both experiments, fast and correct trials were rewarded and slow correct trials were not rewarded; in addition, error trials were penalized in Experiment 2 (but not Experiment 1). We analyzed effects on a global level by assessing N-2 repetition costs in the baseline and reward phase in the reward and control group, as well as on a local level by assessing trial-by-trial reward aftereffects in the reward phase only. Across both experiments, we observed a reward-based modulation of general performance and reliable N-2 repetition costs. However, we did not observe robust effects indicating any reward-related modulation of inhibitory task control.

### Reward-based modulation of task inhibition

The only result suggesting a reward-based modulation of N-2 task repetition costs was found in the drift rate parameter in the local analysis of Experiment 2, in which a strong inhibition of irrelevant task sets was required since errors were penalized. If this effect was robust, it would indicate increased inhibition to currently irrelevant task sets due to recent reward experience. Assuming that effects in drift rate reflect visual attention (Grahek et al., 2021; Wang et al., 2019) and/or strength of task set representations (e.g., Etzel et al., 2016; Hall-McMaster et al., 2019; Yee & Braver, 2018; see also following section), reward experience may locally weaken representations of reward-irrelevant task sets and/or decrease visual attention when switching back to a still-inhibited task after a competing task has been rewarded. However, the Bayes Exclusion Factors and all other analyses across the two experiments suggest the absence of reward-based modulation of task inhibition.

In sum, the evidence for reward-based modulation of task inhibition (as measured by N-2 repetition costs) seems to be inconsistent, ranging from a reward-based *de*crease of task inhibition (Zhang et al., 2016, *N* = 28 per group) to a reward-based *in*crease of task inhibition (Jiang & Xu, 2014; *N* = 20). Our results do not support any modulation even with a larger sample size (two experiments, *N* = 48 per group per experiment). This may help to explain the previously inconsistent findings, suggesting that reward does not interact with cognitive control on the level of task sets at all.

In contrast, the evidence for reward-based modulation of response inhibition reported in the literature seems to be more unequivocal. Several studies investigated the influence of reward on stopping performance in a stop-signal paradigm, by manipulating reward in stop trials (Boehler et al., 2012, 2014; Wang et al., 2019). These studies consistently observed improved stopping performance (i.e., reduced stop-signal reaction time) under reward. Nevertheless, one cannot simply conclude that reward leads to increased response inhibition; rather, the emerging picture is more complex: Wang et al. (2019) empirically disentangled two component processes involved in stop-signal performance: the attentional capture triggered by the stop-signal (mediated by the inferior frontal gyrus (IFG) brain region), and the inhibition of the initiated motor response (mediated by the pre-supplemental motor area (pre-SMA)). They observed that reward improved attentional capture by unexpected stimuli (such as a stop signal), but not stopping of the motor response per se (see also Langford et al., 2016, for reward-related modulation of attention in a stop-signal task). Moreover, when performance in *go* trials is rewarded (instead of performance in stop trials, as in the above-mentioned studies), stop-signal reaction time is *increased* in the reward condition (Padmala & Pessoa, 2010; see Leotti & Wager, 2010; for a theoretical model of reward manipulations on stop-signal performance). Taken together, it seems that reward-based modulations of inhibitory functions are multi-faceted, and more research is necessary for a more complete picture.

### Reward-based modulation of general performance

The present study reveals interesting effects of the reward manipulation on general performance: Participants responded considerably faster, and more error prone, when the reward manipulation was introduced. This performance shift optimized participants’ performance, as it led to a significantly higher reward rate in the reward group relative to the “virtual reward rate” in the control group (where participants had no incentive to shift their performance since no reward was provided). Diffusion modeling revealed that at least two different cognitive processes might be underlying this performance shift in the reward group: Firstly, participants change their response strategy towards faster and less cautious responding, as indicated by a lower boundary separation parameter in the reward phase. Secondly, participants become more focused on the task and have a better signal-to-noise ratio of the currently relevant task representation, as indicated by a higher drift rate in the reward phase.

The observed reward-based performance shift does not constitute a simple speed-accuracy tradeoff. Previous research with instructed speed-accuracy tradeoffs revealed that the reduced boundary separation is typically accompanied by a *decrease* in drift rate and non-decision time in the speed condition relative to the accuracy condition (e.g., Mittelstädt et al., 2022; Rae et al., 2014; Voss et al., 2004). Notably, along with reduced boundary separation, we consistently observed an *increase* in drift rate from baseline to reward phase, which was particularly pronounced in the reward group compared to the control group. Thus, while the participants’ response criterion was more liberal when expecting reward, performance was additionally optimized in terms of drift rate.

The increased drift rate under reward suggests two mutually non-exclusive mechanisms: Firstly, visual attention likely increases under reward prospect as we used visual stimuli in all trials (see Grahek et al., 2021, for EEG evidence that reward enhances stimulus processing in the visual cortex; Wang et al., 2019). Secondly, mental representations of task sets are likely improved due to better signal-to-noise ratio under reward as we used N-2 task switching paradigms in both experiments (Etzel et al., 2016; Hall-McMaster et al., 2019; Yee & Braver, 2018).

Since every trial was a task switch in the present study, we cannot determine whether the process of switching between tasks was facilitated, or whether the better signal-to-noise ratio was independent from switching and would occur on task repetition trials as well. On a more general level, the reward-triggered increase in drift rate is consistent with the notion of sharpened task set representations under reward, which is presumably mediated by a dopamine-based enhancement of attentional focus under reward (e.g., Aarts et al., 2010; Durstewitz & Seamans, 2002, 2008; Goschke & Bolte, 2014; Yee & Braver, 2018). There is neuroscientific evidence that reward leads to an improved signal-to-noise ratio of task set representations. For instance, an fMRI study on task switching using the methodology of multivariate pattern analysis (MVPA) showed that task set representations in frontoparietal brain regions become more discriminable under reward (Etzel et al., 2016). Converging evidence comes from a recent EEG study using representational similarity analysis, which found that task-set representations were enhanced under reward, and this reward-based enhancement was more pronounced on task switch trials than task repetition trials (Hall-McMaster et al., 2019). As the increase in drift rate is associated with the onset of reward expectancy in the present study, we would expect similar results with increasing reward expectations on a local trial-by-trial basis (e.g., Fröber et al., 2018, 2019, 2020; Fröber & Dreisbach, 2016a, 2021).

### Reward expectancy

In research on motivation and reward, different aspects of reward have been distinguished (Berridge & Robinson, 2003), which in turn have differential impact on cognitive control functions (Notebaert & Braem, 2015). Berridge and Robinson (2003) distinguished between three components of reward: the “motivational component” (i.e., reward expectancy), the “affective component” (i.e., the positive affect associated with reward), and the “learning component” (i.e., reinforcement learning, where rewarded behaviors are more likely to be executed again). Building up on this distinction, Notebaert and Braem (2015) suggested that each reward component has a distinct impact on cognitive control functions: The motivational component of reward (i.e., reward expectancy) promotes anticipatory behavior, or “proactive control” (as opposed to “reactive control”) in the dual-mechanisms of control (DMC) framework by Braver (2012). The affective component of reward (i.e., positive affect) promotes cognitive flexibility and explorative behavior. Finally, the learning component of reward promotes the repeating of actions that have been rewarded in the past.

The reward effects observed in the present study are most likely related to the motivational component (i.e., reward expectancy), rather than to the affective and learning components of reward processing. We observed global effects of reward, that is, faster RTs and higher error rates in the reward phase of the reward group, together with higher drift rate and lower boundary separation in this phase. Moreover, we investigated local reward aftereffects in the reward phase. We observed faster RTs, higher drift rate and lower boundary separation after rewarded trials than after non-rewarded trials in the reward group. Importantly, we observed the same data pattern also in the control group, when splitting the data into fast correct trials (that would have been rewarded in the reward group) and slow correct trials (that were not rewarded). This suggests that the global reward effects observed in the present study are more likely due to the prospect of receiving a reward in the upcoming trial (i.e., reward expectancy), rather than the experience of reward in the preceding trial. Hence, the reward effects observed in the present study are probably related to the motivational component of reward expectancy, and less so to the affective and learning components of reward processing.

It should be noted that in the present study, every trial in the reward phase could potentially be rewarded. Hence, the prospect of reward was constant across all trials in the reward phase (but differed between reward and baseline phase, and between the groups; see Zhang et al., 2016, for a similar design). This is different from other studies that manipulated reward expectancy on a trial-by-trial basis (e.g., Hippmann et al., 2019; Jiang & Xu, 2014). In these studies, participants received a cue at the beginning of each trial indicating whether a large or small/zero reward could be gained in the upcoming trial. Given that reward expectancy seemed to be the component that was most likely driving the reward effects in the present study, future research might benefit from incorporating a trial-by-trial manipulation of reward expectancy. Such a manipulation would also allow for analyzing the influence of changes in reward expectation, which have been shown to modulate task-switching performance (e.g., Fröber & Dreisbach, 2016b). Regarding N-2 repetition costs, it is conceivable that an increasing reward expectation from trial N-2 to trial N-1 (relative to a high-remaining reward expectation from N-2 to N-1) might modulate N-2 repetition costs. Assuming that an increasing reward expectation increases cognitive flexibility and leads to smaller task switch costs and increased voluntary task switch rates (Fröber et al., 2018, 2019, 2020; Fröber & Dreisbach, 2016b, 2021; Shen & Chun, 2011), one might hypothesize that less task inhibition is necessary, and hence, N-2 repetition costs become smaller when reward expectancy increased from the N-2 trial to the N-1 trial.

### Limitations

Two concerns may be raised questioning the effectiveness of our between-group reward manipulation: Firstly, between-group differences in (virtual) reward scores were numerically much smaller in Experiment 2 than in Experiment 1; and secondly, behavioral data showed decreasing RTs and increasing error rates from the baseline to the reward phase in *both* groups, which may be interpreted as a speed-accuracy tradeoff elicited by the verbal feedback rather than the point-based reward scheme. Regarding the first concern, the reward group obtained statistically significantly higher reward scores than the control group. The numerically small between-group difference in Experiment 2 was likely due to the different reward scheme in which participants in the reward group could actually loose points. Regarding the second concern, we acknowledge that drift rate increased from baseline to reward phase in both groups. However, in both experiments, this effect was significantly larger in the reward group than in the control group, which can only be explained by reward expectancy specific to the point-based reward scheme in the reward group. As discussed above (see section on *Reward-based modulation of general performance*), the present data cannot be explained by a simple speed-accuracy tradeoff, but rather it indicate a reward-specific optimization of performance.

Significant N-2 task repetition costs were observed in RT and drift rate. While they were numerically small (21 ms in both experiments), they were statistically significant in all three task switching paradigms (face and digit categorizations in Experiment 1; categorizations of household items in Experiment 2), and hence can be considered robust. Nevertheless, should reward modulate N-2 repetition costs, it would be easier to find corresponding interaction effects if N-2 repetition costs were larger.

While our overall result pattern and Bayesian analyses suggest that inhibitory control as measured by N-2 repetition costs is not modulated by reward, we found one significant result indicating a local reward-based increase of inhibition to reward-irrelevant task sets in the diffusion model drift rate parameter. This was found in Experiment 2, where the reward scheme penalized errors to strenghten the requirement for inhibitory control. Thus, it may be speculated that stronger reward manipulations could be necessary to detect a robust reward-based modulation of task-set inhibition.

In sum, we encourage a stronger reward manipulation for future research to allow for drawing stronger conclusions about the absence – or presence – of reward-based modulation of task-set inhibition. This may be accomplished by setting the reward threshold even faster, while maintaining point withdrawal for incorrect answers or even increasing it (e.g., withdrawal of *two* points for each error). This would also prevent or reverse the high focus on speed leading to floor effects in RT.

### Conclusion

In the current task-switching study with reward manipulation, we did not observe any robust evidence for reward-related modulation of N-2 repetition costs, raising the possibility that reward does not interact with inhibitory control on the task level. At the same time, the reward manipulation had pronounced effects on general performance, with faster and more error-prone performance in the reward group than control group. Diffusion modeling revealed that the reward manipulation induced a reduction in the boundary separation parameter, indicating a shift towards a less cautious response strategy under reward, as well as an increase in the drift rate parameter, consistent with the notion of sharpened task-set representations under reward, which are mediated by the dopamine system.

## Data Availability

The raw data and additional material are available at: https://osf.io/uyxc4.

## Footnotes

1 Jiang and Xu (2014) also analyzed N-2 repetition costs as a function of the N-2 incentive cue, and observed smaller N-2 repetition costs after N-2 incentive (vs. no-incentive) cues. However, in their study the sequence of incentive cues was constrained in such a way that an incentive cue in one trial was always followed by a no-incentive cue in the subsequent trial, but not vice versa, making the interpretation of triplet sequences difficult.

2 For one participant in the digit task control group, the computer program crashed during the fourth block, such that the last 83 trials were not collected from this participant.

3 We also checked for potential differences between the two paradigms (digit tasks vs. face tasks) by performing an analysis with paradigm as an additional between-subject variable, which is reported as additional material in the Open Science Framework (OSF), see Part I (Table A1, Fig. A1), https://osf.io/uyxc4/?view_only=f671a29d9ef34603bd7cf6fe1d9b595c. We did not find any relevant differences regarding reward-based modulation of N-2 task repetition costs.

4 We applied two different data filtering criteria for analysis of mean performance and diffusion model analysis in order to maximize comparability of our results with the respective literature for behavioral studies and diffusion model studies.

5 One possible reason for the overall small N-2 repetition costs in the present experiment is that performance-contingent positive feedback was provided after correctly responded trials throughout the experiment, which might have acted as a reward signal and attenuated N-2 repetition costs. However, N-2 repetition costs were very similar between the primary control group and an additional control group without such positive feedback after correct trials, speaking against this possibility (see OSF Additional Material: Part III, Tables A2 and A3).
